# PCV13 vaccine prevents pneumococcal biofilms without affecting *Staphylococcus aureus* population within the polymicrobial biofilm

**DOI:** 10.3389/fimmu.2024.1495932

**Published:** 2024-11-01

**Authors:** Julio Sempere, José Yuste, Mirian Domenech

**Affiliations:** ^1^ Spanish Pneumococcal Reference Laboratory, National Center for Microbiology, Instituto de Salud Carlos III, Madrid, Spain; ^2^ CIBER de Enfermedades Respiratorias (CIBERES), Instituto de Salud Carlos III, Madrid, Spain; ^3^ Department of Genetics, Physiology and Microbiology, Faculty of Biology, Complutense University of Madrid, Madrid, Spain

**Keywords:** PCV13, biofilms, OPA, *S. aureus*, MRSA, *S. pneumoniae*

## Abstract

In respiratory pathogens such as *Streptococcus pneumoniae*, biofilm formation is associated with the colonization of the nasopharynx and chronic respiratory infection. Previous data have shown that pneumococcal conjugate vaccines (PCVs) had an impact on *S. pneumoniae* colonization and a potential replacement by other respiratory pathogens such as *Staphylococcus aureus*. The objective of this work was to evaluate the evasion of the immune system by monospecific biofilms and by *S. aureus-S. pneumoniae* mixed biofilms. We performed opsonophagocytosis assays (OPA) using human HL-60 against previously disaggregated monospecific biofilms of MSSA, MRSA and *S. aureus-S. pneumoniae* mixed biofilms. We used pre-immune and post-immune serum from immunocompetent adult patients vaccinated with PCV13. Immune sera had a clear effect in reducing pneumococcal biofilms of serotypes 3, 14, 18C, 19F and 19A, whereas had no effect in non-PCV13 serotypes such as 8, 11A and 24F. Our study confirmed that serum from vaccinated patients with PCV13 did not have any effect in reducing *S. aureus* population in monospecific biofilms, regardless the methicillin resistance phenotype. Moreover, immunized sera from vaccinated patients with PCV13 did not have any effect in *S. aureus* population in the mixed biofilm, whereas significantly reduced the population of pneumococcal serotype 19A strain in the mixed biofilm which is of great interest because this serotype is included in PCV13, and it is associated with vaccine failures.

## 1 Introduction

Chronic obstructive pulmonary disease (COPD) and cystic fibrosis (CF) are progressive inflammatory pathologies affecting the lungs characterized by impaired mucociliary clearance, mucus hypersecretion, and altered mucosal immunity. These factors contribute to the colonization of the lower respiratory tract by different bacterial species. The presence of bacterial biofilms is very common in these two chronic pulmonary diseases as well as in acute otitis media or even during the carriage state (1, 2). *Streptococcus pneumoniae*, or pneumococcus, is a common pathogen causing these pathologies, being responsible for many of the acute exacerbations and chronic bronchiectasis in these patients (2). Polymicrobial biofilm development is a usual occurrence in persistent respiratory infections, allowing bacterial communities to evade host immune responses and enhance antimicrobial resistance. The pneumococcal biofilm demonstrates an enhanced ability to evade the host immune response, specifically by reducing complement system activation and avoiding phagocytosis (3). Compared to planktonic bacteria, biofilm formation is associated with a decreased deposition of complement components such as C3 and C1q, and increased recruitment of the regulatory protein factor H on the bacterial surface. Confocal microscopy analysis revealed that C3 molecules could permeate all layers of the intact biofilm and exposure to serum components do not affect its architecture. The observed differences in complement deposition are attributed to phenotypic alterations in pneumococcal cells within the biofilm, persisting even after biofilm disaggregation (3).

The use of pneumococcal conjugate vaccines (PCVs) has shown to be effective in reducing the burden of disease and the carrier state by vaccine serotypes, but long-term use may lead to serotype replacement by non-vaccine serotypes (4, 5). Additionally, some PCV13 vaccine serotypes have still a high burden of disease despite the introduction of the vaccine, being serotype 3 the most frequent followed by serotypes 19A, 14 and 19F (5–9). This is attributed to a moderate impact of PCVs against pediatric colonization (10). Since the introduction of PCV7, epidemiological studies have shown contradictory reports on the interaction between conjugate vaccines, pneumococcus, and *Staphylococcus aureus.* Some reports show a detrimental effect describing increase colonization and disease rates by *S. aureus* in vaccinated population (11–14) whereas other reports show no effect (15, 16) or even a beneficial contribution indicating that vaccination with PCV13 in children diminished *S. aureus* colonization in both the nasopharyngeal tract and the middle ear. (17, 18) The purpose of this report was to demonstrate for the first time that vaccination with PCV13 has effect against pneumococcal serotypes forming biofilms and the potential impact on polymicrobial biofilms including methicillin resistant *S. aureus* and *S. pneumoniae*.

## 2 Materials and methods

### 2.1 Strains and human sera

Pneumococcal strains used belonged to PCV13 serotypes (S3, S14, S18C, S19A, S19F) and non-PCV13 serotypes (S8, S11A, S24F) (**Table 1**). For *S. aureus* monospecific biofilms, we used the Methicillin-Sensitive *S. aureus* (MSSA) and Methicillin-Resistance *S. aureus* (MRSA) strains whereas for mixed biofilms we used the MRSA strain and a pneumococcal serotype 19A strain (**Table 1**). Human sera (pre-immune or post-PCV13 1 month after vaccination) were obtained from seven healthy immunocompetent adults with an age range of 25-50 years old and a gender distribution of 57% females and 43% males. All participants provided written informed consent (authorization approval of Ethics Committee: HULP: PI-1832). The project was approved by ISCIII Ethics Committee (Ref: CEI PI 45_2021-v2).

**Table 1:** Strains used in the study.

Strain	Species	Serotype/Description	Reference/Origin
**69/08**	*S. pneumoniae*	19F/Blood; adult; pneumonia	SPRL
**608/20**	*S. pneumoniae*	14/Blood; pediatric; pneumonia	SPRL
**854/12**	*S. pneumoniae*	18C/Blood; adult; pneumonia	SPRL
**1090/20**	*S. pneumoniae*	14/Blood; pediatric; pneumonia	SPRL
**1228/19**	*S. pneumoniae*	19A/Blood; adult; pneumonia	SPRL
**1732/19**	*S. pneumoniae*	11A/Blood; adult; pneumonia	SPRL
**1734/19**	*S. pneumoniae*	8/Blood; adult; pneumonia	SPRL
**1743/19**	*S. pneumoniae*	3/Blood; pediatric; pneumonia	SPRL
**2291/19**	*S. pneumoniae*	18C/Blood; pediatric; bacteremia	SPRL
**3017/13**	*S. pneumoniae*	24F/Blood; pediatric; pneumonia	SPRL
**60031/19**	*S. aureus*	5/MSSA; blood; adult	(19)
**60061/19**	*S. aureus*	5/MRSA; wound exudate; adult	(19)

SPRL, Spanish Pneumococcal Reference Laboratory.

### 2.2 Biofilm formation

Pneumococcal monospecific biofilms of *S. pneumoniae*, *S. aureus* (MSSA and MRSA) or mixed biofilms of both bacterial species were developed following recent protocols described in our laboratory (19). Briefly, cells were grown in a C+Y medium to an *A*_550_ of ≈ 0.5-0.6 and diluted 100-fold. *S. pneumoniae* and *S. aureus* suspensions were used individually or 1:11 inoculum proportion mixed biofilms (MRSA: *Sp*, 1:11, to obtain 1:1 viability in the mature mixed biofilm), and aliquots of 200 µL were added into 96-well polystyrene treated plates. The biofilms were incubated from 5 h at 34°C. After incubation, the planktonic culture was separated, and biofilms were gently disaggregated using a pipette to serve as inoculum of opsonophagocytosis assays (OPA).

Dilutions were culture on blood agar plates and CFU/ml were determined the next day after incubation. In mixed infections, we used blood agar plates with 5 µg/ml of gentamicin and plates containing Salt Mannitol Agar to select *S. pneumoniae* and *S. aureus* respectively.

### 2.3 Opsonophagocytosis assays

OPA were performed using human HL-60 cells (CCL-240;ATCC) differentiated to granulocytes as previously described (20, 21). In brief, we used 10^5^ HL-60 cells differentiated to granulocytes and 2.5 × 10^2^ CFU of *S. pneumoniae*, *S. aureus* or both (mixed biofilms) [multiplicity of infection (MOI) 400:1] that were previously opsonized with HBSS (control) or 1/8 of heat-inactivated pre-immune or immune sera. If there were no differences between the OPA result from pre-immune sera compared to control, we consider that there are not natural anti-*Streptococcus* or anti-*Staphylococcus* functional antibodies in the immunocompetent subjects tested. The limit of ≥50% killing was considered as a positive response eliciting functional antibodies (20, 21). In addition, pre-immune or immune sera were tested against disaggregated biofilms and against planktonic cultures of the different strains to confirm the functional activity of immune sera.

### 2.4 Confocal laser scanning microscopy of biofilms

We used CLSM to visualize the cells as previously described (19). Briefly, biofilms were grown as explained above on glass-bottomed dishes (WillCo-dish; WillCo Wells B.V., The Netherlands) for 5 h at 34°C (19). Non-adherent bacteria were removed from biofilms by washing with sterile water, and bacterial viability within the biofilm was assessed using the LIVE/DEAD BacLight kit (Invitrogen). CLSM observations were made with a Leica spectral SP5 confocal microscope and analyzed with the LAS AF software. Images represent the x–y from XYZ-stacks at 0.5-µm intervals.

### 2.5 Statistical analysis

Data were obtained from different independent experiments, containing at least three replicates in each experiment. To obtain OPA (%), viability data (CFU/ml) was normalized to control (OPA with HBSS and the source of complement). A two-tailed Student’s t test was used for two groups’ comparison. Statistical analysis were performed using GraphPad 8.0 software. Each serotype was tested against at least three different patient’s sera.

## 3 Results

**Figure 1:**
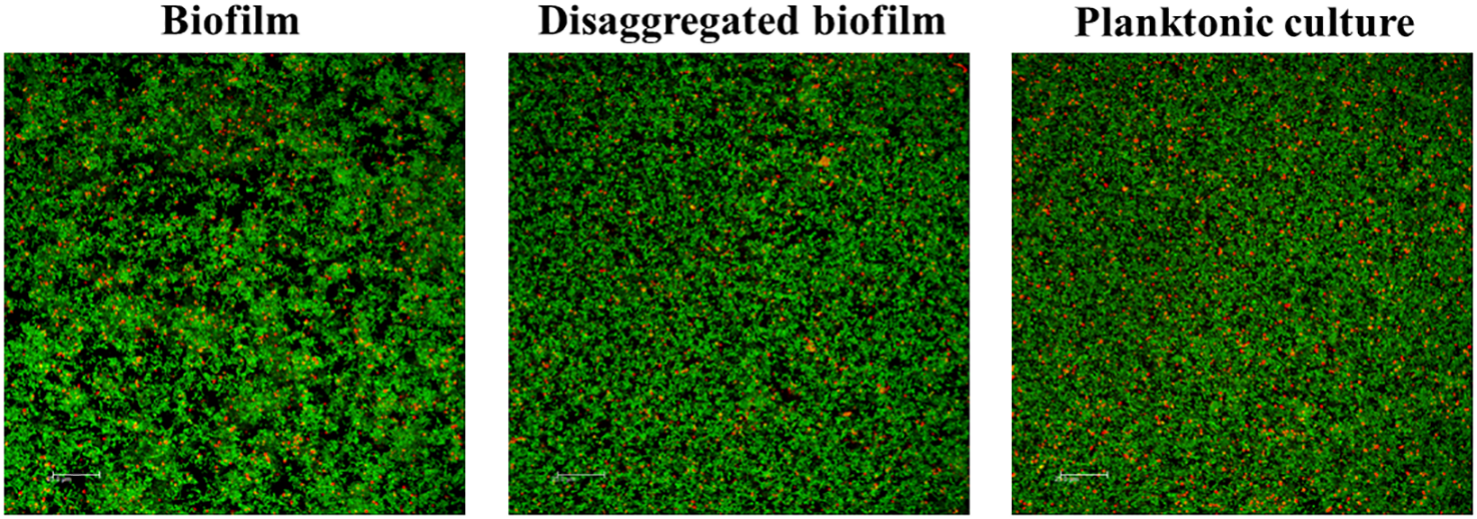
Architecture of a biofilm, disaggregated biofilm and planktonic culture of *S. pneumoniae* before adding vaccine serum. Biofilms and planktonic culture were stained with the BacLight kit (Invitrogen) to reveal viable (green fluorescence) and non-viable (red fluorescence) bacteria. Scale bars, 25 μm.

**Figure 2:**
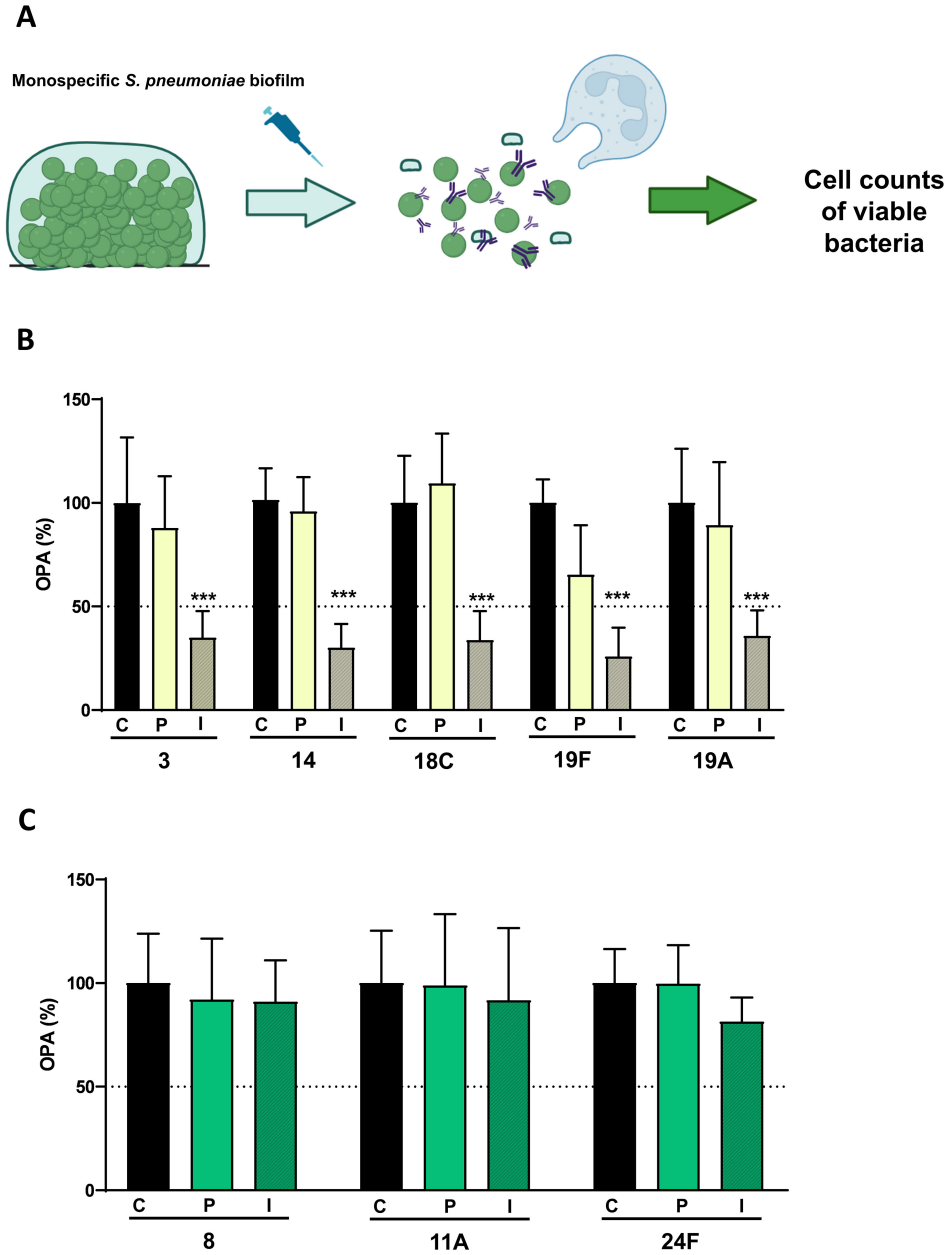
Opsonophagocytosis killing assays (OPA) of pneumococcal biofilms of different serotypes mediated by PCV13. **(A)** Scheme for the OPA assay of pneumococcal cells forming the biofilm. Briefly, after incubation, the biofilm was gently disaggregated with a pipette. Later, disaggregated cells were exposed to complement and HBSS (control), pre-vaccine (pre-immune) or immune serum (1/8 dilution), and to HL-60 neutrophils in a 400:1 MOI (HL-60: bacteria). **(B)** OPA (%) of pneumococcal serotypes included in PCV13 vaccine. **(C)** OPA (%) of pneumococcal serotypes not included in PCV13 vaccine. Black bars show biofilm control of different serotypes in the absence of human serum, with HBSS and the source of complement (C: Control). To obtain OPA (%), viability data (CFU/ml) were normalized to control (OPA with HBSS and the source of complement).Open bars show biofilms of different serotypes incubated with human non-vaccine serum (P: pre-vaccine serum). Hatched bars show biofilms of different serotypes incubated with serum from vaccinated individuals (I: Immune vaccine serum). Diagrams were made with BioRender. Columns represent means and standard deviation bars are shown, and asterisks mark statistically significant results (two-tailed Student’s t test: ***P < 0.001) when comparing the biofilm exposed to immune sera *versus* the control.

We first evaluated the morphology of *S. pneumoniae* when forming biofilms. Bacteria in a planktonic state and in a disaggregated biofilm state prior to serum addition exhibited a similar distribution or aggregation (**Figure 1**). Phenotypic biofilm resistance to the immune system was previously demonstrated to be equal in intact and disaggregated biofilms (3). Exposure of pneumococcal monospecific biofilms to human pre-immune sera did not show any killing effect compared to control (**Figures 2A, B**). However, using sera from PCV13 vaccinated individuals (immune sera), we observed a significant biofilm reduction for all the vaccine serotypes analyzed. These results confirm that PCV13 vaccination elicits functional antibodies that induce opsonophagocytosis of pneumococcal biofilms by vaccine serotypes (**Figure 2B**). In the case of biofilms by non-vaccine serotypes, immune sera did not reduce the pneumococcal biofilm demonstrating that the vaccine is effective only against the biofilms of vaccine-covered serotypes as expected (**Figure 2C**).

**Figure 3:**
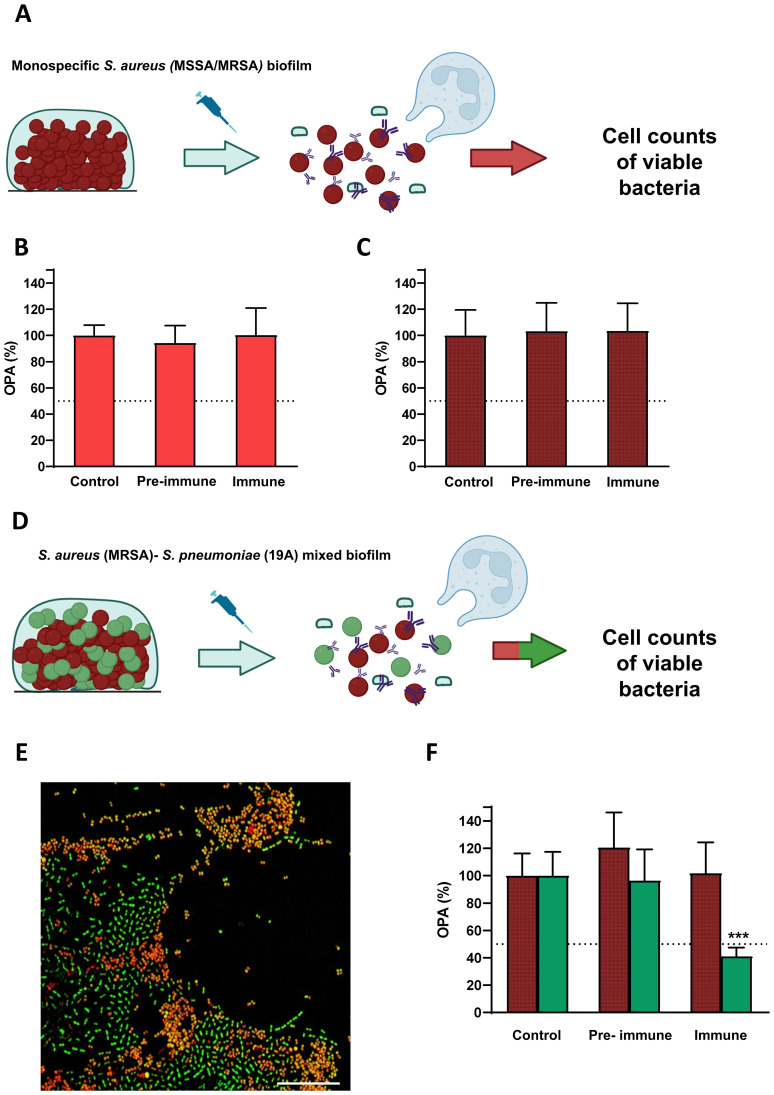
Opsonophagocytosis killing assays (OPA) of *S. aureus* monospecific biofilms or mixed biofilms containing *S. pneumoniae* mediated by PCV13. **(A)** Scheme for the OPA assay of *S. aureus* cells forming the monospecific biofilm. Briefly, after incubation, the biofilm was gently disaggregated with a pipette. Later, disaggregated cells were exposed to complement and HBSS (control), pre-vaccine (pre-immune) or immune serum (1/8 dilution), and to HL-60 neutrophils in a 400:1 MOI (HL-60: bacteria). **(B)** OPA of MSSA monospecific biofilms. **(C)** OPA of MRSA monospecific biofilms. **(D)** Scheme for the OPA assay of the mixed biofilm containing MRSA and *S. pneumoniae* of serotype 19A. Briefly, the mixed biofilm of MRSA-*S. pneumoniae* was incubated following previous methodology (19). Then, biofilm was gently disaggregated with a pipette. Later, disaggregated cells were exposed to complement and PBS (control), pre-vaccine (pre-immune) or immune serum (1/8 dilution), and to HL-60 neutrophils in a 400:1 MOI (HL-60: bacteria). **(E)** Confocal microscopy image showing *S. aureus* in red and *S. pneumoniae* in green using the BacLight kit (Invitrogen) component B Scale bars, 25 μm. **(F)** OPA assay of the mixed biofilm containing MRSA in red hatched bars and *S. pneumoniae* of serotype 19A in green bars. To obtain OPA (%), viability data (CFU/ml) were normalized to control (OPA with HBSS and the source of complement). Diagrams were made with BioRender. Columns represent means and standard deviation are shown, and asterisks mark statistically significant results (two-tailed Student’s t test: ***P < 0.001) when comparing the biofilm exposed to immune sera versus the control.

To evaluate the possible heterologous effect of PCV13 against other respiratory pathogens that colonize the upper respiratory tract such as *S. aureus*, we tested pre-immune sera or immune sera from patients vaccinated with PCV13 against the monospecific biofilm of MSSA and MRSA strains (**Figures 3A–C**). The level of the biofilm remained unchanged regardless of the sera used indicating that antibodies elicited against PCV13 did not have any impact on the *S. aureus* biofilm (**Figure 3B, C**).

In addition, we also evaluated the impact of PCV13 against a mixed biofilm formed by a MRSA strain and a multidrug-resistant strain of serotype 19A of pneumococcus (PCV13 serotype) (**Figures 3D, E**). Hence, we confirmed that vaccination with PCV13 did not have any effect against *S. aureus* in the polymicrobial biofilm whereas significantly reduced the pneumococcal population within the mixed biofilm (**Figure 3F**). Moreover, *S. aureus* population cells within the mixed biofilm did not increase when *S. pneumoniae* was killed in the presence of immune sera suggesting that PCV13 vaccination only targets pneumococcal cells of vaccine-covered serotypes without inducing replacement by MRSA of the niche left by pneumococcus (**Figure 3F**).

## 4 Discussion

Colonization of the upper respiratory tract is essential for the pneumococcal pathogenesis process as in many cases the bacterium can disseminate producing non-invasive diseases (acute otitis media, sinusitis, non-bacteraemic pneumonia) or even invasive pneumococcal disease (IPD) (22). The use of PCVs is the best prophylactic strategy to prevent the development of these diseases reducing the morbidity and mortality rates (4). Immunogenicity studies evaluating PCVs in children and adults are always based on their ability to induce functional antibodies that trigger the opsonophagocytosis process of the bacterium. The contribution of PCVs in the reduction of AOM, bronchiectasis, hospitalizations of COPD and even the carriage state is well known and in all these conditions and pathologies, the presence of biofilms is a generic event (1, 2). However, there are no studies demonstrating that antibodies elicited after vaccination with PCVs have the potential to recognize and induce the clearance of bacteria forming the biofilm. In pneumococcal pathogenesis, pediatric isolates are better biofilm formers than adult isolates and emerging serotypes causing IPD in adults can originally be from strains colonizing the pediatric nasopharyngeal tract and therefore, prevention of the carrier state may be a very useful strategy to induce herd protection in adults (23).

The *in vitro* biofilm model described in this study may be a good representation of an airways biofilm. In this sense, biofilms covering multiwell plates have proven to be a good platform for testing antimicrobial or antibiofilm compounds, both for dispersing and preventing biofilm formation (24). Similar results have been obtained when comparing antimicrobial treatments using *in vitro* biofilms and murine models of pneumonia (25). *In vitro* biofilms are also a valuable tool for predicting emerging serotypes or genotypes associated with colonization (26, 27). Finally, this *in vitro* biofilm system is a good platform for studying interactions between microorganisms that constitute the same biofilm (19).

Our results using an *in vitro* biofilm platform, human sera from vaccinated individuals and neutrophils derived from HL-60 cells, confirm for the first time that PCV13 is immunogenic against pneumococcal biofilms formed by vaccine-preventable serotypes including serotype 3. This is in agreement with epidemiological studies demonstrating that the introduction of PCV13 in pediatric immunization programs has reduced up to 38% of all IPD cases in children (4). Moreover, the introduction of PCV13 diminished the IPD cases associated with antimicrobial resistance such as those caused by serotypes 19F and 19A (9), that are also associated with the biofilm state (28). Our results are of great interest for public health because we demonstrate for the first time a beneficial preventive effect of PCV13 elicited antibodies against the biofilm state of both serotypes. Despite observing that immunized sera from adult patients provided protection against biofilms formed by the vaccine serotypes tested, the high prevalence of some of them such as serotype 3 in the adult population (5) can largely be attributed to insufficient vaccine coverage in adults (4, 5, 29).

The impact of PCVs on the nasopharyngeal niche is concerning, as the reduction in pneumococcal colonization could create an opportunity for other invasive pathogens to occupy the space left by the pneumococcus. Additionally, non-sterilizing immunization may be beneficial in preventing colonization by new pathogens that could cause similar or more severe diseases (30, 31). In terms of heterologous protection, there was no effect against *S. aureus* but antibodies to PCV13 reduced significantly the pneumococcal population within the polymicrobial biofilm. These results could shed light on the discrepancies found in literature, as different studies, mainly focus on carriage in children vaccinated or not with PCVs, have obtained different outcomes when evaluating the changes in *S. aureus* carriage due to PCVs (15–17). Our results align with previous studies demonstrating that the use of PCVs is effective in pneumococcal nasopharyngeal carriage in children suffering AOM without any change in *S. aureus* carriage (15–17).

## Data Availability

The raw data supporting the conclusions of this article will be made available by the authors, without undue reservation.
